# Phytochemical Compounds and Protection from Cardiovascular Diseases: A State of the Art

**DOI:** 10.1155/2015/918069

**Published:** 2015-10-04

**Authors:** Beniamino Pagliaro, Caterina Santolamazza, Francesca Simonelli, Speranza Rubattu

**Affiliations:** ^1^Department of Clinical and Molecular Medicine, School of Medicine and Psychology, Saint Andrea Hospital, Sapienza University of Rome, 00189 Rome, Italy; ^2^IRCCS Neuromed, 86077 Pozzilli, Italy

## Abstract

Cardiovascular diseases represent a worldwide relevant socioeconomical problem. Cardiovascular disease prevention relies also on lifestyle changes, including dietary habits. The cardioprotective effects of several foods and dietary supplements in both animal models and in humans have been explored. It was found that beneficial effects are mainly dependent on antioxidant and anti-inflammatory properties, also involving modulation of mitochondrial function. Resveratrol is one of the most studied phytochemical compounds and it is provided with several benefits in cardiovascular diseases as well as in other pathological conditions (such as cancer). Other relevant compounds are *Brassica oleracea*, curcumin, and berberine, and they all exert beneficial effects in several diseases. In the attempt to provide a comprehensive reference tool for both researchers and clinicians, we summarized in the present paper the existing literature on both preclinical and clinical cardioprotective effects of each mentioned phytochemical. We structured the discussion of each compound by analyzing, first, its cellular molecular targets of action, subsequently focusing on results from applications in both ex vivo and in vivo models, finally discussing the relevance of the compound in the context of human diseases.

## 1. Introduction

Cardiovascular diseases (CVDs) still remain the primary cause of death worldwide according to the World Health Organization and American Heart Association statistics [1]. Different approaches have been proposed to reduce the high global incidence of CVDs and to improve human health. The consumption of functional foods or dietary supplements for lowering the risk of CVDs has gained attention over the last few years from both scientific and clinical communities [2, 3]. Several antioxidant compounds can be found in vegetables (e.g., vitamins and phenolic compounds). They are partly responsible for the health benefits by scavenging reactive oxygen radicals (ROS) and by inhibiting cellular damage at different levels. Although the literature contains several review articles describing either general health benefits of phytochemical supplements or the cardioprotective effects of a single phytochemical compound, no comprehensive review article has been so far reported focusing on both preclinical and clinical cardiovascular beneficial effects of the most known compounds (resveratrol,* Brassica oleracea*, curcumin, and berberine).

In the present paper we attempted to fill up this literature gap. In order to reach our goal, we discussed each phytochemical compound by analyzing its molecular targets of action, discussing all existing in vitro, ex vivo, and in vivo data related to its cardiovascular beneficial properties, finally highlighting the evidence available in human CVDs.

## 2. Resveratrol

Resveratrol (3,5,4′-trihydroxy-trans-stilbene) is a natural polyphenolic compound that exists in* Polygonum cuspidatum*, grapes, peanuts, and berries, as well as in their manufactured products, especially red wine [4]. It exists in both* cis*- and* trans*-configurations, of which* trans*-resveratrol is the principal biologically active form [5]. Interestingly, red wine (therefore resveratrol) was supposed to be one of the factors responsible for the “French Paradox,” together with several lifestyle and dietary factors. The term is used to describe the low incidence of CVDs in French population despite its high intake of saturated fats. However, the median daily dose of resveratrol to be protective is estimated to be 20 mg/kg/day, whereas its concentration in red wine is roughly 1.98–7.13 mg/L [6]. Therefore, the assumption of a high quantity of red wine per day would be needed for a man to obtain a protective dose of resveratrol, causing serious health problems [7, 8]. However, an inverse association between moderate alcohol consumption (30–50 gr/day) and CVDs has been assessed in several epidemiological studies. The physiological mechanism of the protective effect of alcohol seems to be at least in part related to its effect in reducing platelet action.

### 2.1. Molecular Targets and Properties

**Figure 1:**
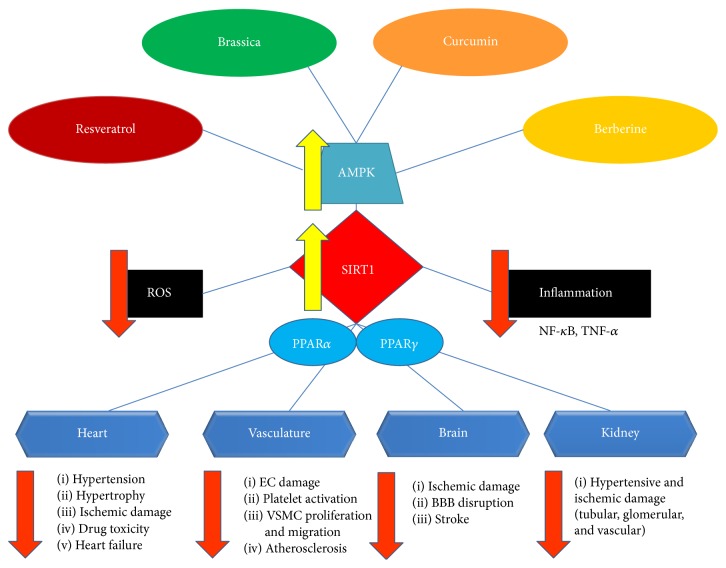
Schematic representation of beneficial effects exerted in the cardiovascular system by resveratrol,* Brassica oleracea*, curcumin, and berberine. The AMPK/SIRT-1/PPAR*α*/*γ* molecular pathway, underlying most of the effects of all vegetable compounds, is illustrated. AMPK: 5′-adenosine monophosphate-activated protein kinase; SIRT-1: silent mating type information regulation-1; PPAR: peroxisome proliferator-activated receptor; ROS: reactive oxygen species; NF-*κ*B: nuclear factor-*κ*B; TNF-*α*: tumor necrosis factor-*α*; EC: endothelial cell; VSMC: vascular smooth muscle cell; BBB: blood brain barrier.

Resveratrol interacts with multiple targets in cardio- and cerebrovascular diseases, age-related diseases, cancer, and so forth, [9, 10]. The main molecular mechanism mediating resveratrol biological effects is the 5′-adenosine monophosphate-activated protein kinase (AMPK)/silent mating type information regulation-1 (SIRT-1) pathway (Figure 1) [11, 12]. The precise mechanism through which resveratrol activates SIRT-1 is not completely understood [13]. Other minor pathways mediate some of the resveratrol effects and they will be briefly mentioned below.

The most important properties of resveratrol are connected with oxidative stress, vascular inflammation, and platelet aggregation. In fact, resveratrol upregulates the endogenous antioxidant systems, such as superoxide dismutase (SOD) enzymes, in endothelial cells (ECs) and in cardiac myoblasts and it reduces ROS production [14, 15]. Moreover, it reduces arachidonic acid and prostaglandin E2 synthesis. It inhibits phospholipase A2 and ciclooxygenase-2 activity; it antagonizes the function of the most important molecules involved in inflammation, such as nuclear factor-*κ*B (NF-*κ*B), tumor necrosis factor-*α* (TNF-*α*), interleukin-6 (IL-6), inducible nitric oxide synthase (iNOS) activity, and monocyte chemotactic protein-1 (MCP-1) [16–19]. Resveratrol also prevents platelet activation by modulating platelet adhesion, secretion and activation signaling, ROS production, and apoptosis and by enhancing nitric oxide (NO) production [20–22]. Furthermore, several studies demonstrated that resveratrol inhibits protein kinase C (PKC) activation and intracellular calcium release, thus blocking phosphoinositide metabolism upstream platelet activation signaling [23].

### 2.2. Hypertension: Preclinical Studies

The “antihypertensive” effect of resveratrol is thought to be mediated by both endothelium-dependent and endothelium-independent mechanisms [24, 25], by inhibition of vascular smooth muscle cell (VSMC) contractility, by reduction of vasoconstrictor molecules expression (angiotensin (Ang) II and endothelin (ET)-1), and by inhibition of strain-induced ET-1 gene expression through the extracellular-signal regulated kinase (ERK) 1/2 pathway [11, 26, 27]. Finally, its effect on the sympathetic nervous system (SNS) can also contribute to blood pressure (BP) lowering [28]. The antihypertensive effect of resveratrol (administered at the dose of 10–320 mg/kg body weight/day) has been demonstrated, although with some controversial results, in several hypertensive animal models, including spontaneously hypertensive (SHR), two kidney one-clip hypertensive, partially nephrectomized, and deoxycorticosterone acetate- (DOCA-) salt hypertensive rat models, and in the Ang II-infused mouse [11, 29–32]. Controversies appear mainly related to the specific model under study. In particular, resveratrol was more effective in lowering BP in animals with either diabetes or metabolic syndrome in which variable doses of resveratrol (20 mg/kg/day) were administered [33].

Along with the antihypertensive effect, an improvement of endothelial function was described, being largely attributable to endothelial NO synthase (eNOS) activation [34, 35]. This effect can certainly contribute to protecting vasculature from hypertensive damage [36].

#### 2.2.1. Hypertension: Clinical Studies

Scarce information is available regarding the antihypertensive effect of resveratrol in humans. A recent meta-analysis has shown that treatment with ≥150 mg/day of resveratrol, considered as a very low dose, decreases systolic BP (SBP) without affecting diastolic BP (DBP) [37]. Interestingly, doses of 12,5–100 *μ*L of resveratrol were shown to enhance Acetylcholine-mediated vasorelaxation in blood vessels from patients with hypertension and dyslipidemia but not in vessels from healthy subjects [38]. Although resveratrol supplementation did not exert any effect on BP in healthy obese adults and in patients with metabolic syndrome, it significantly improved flow-mediated dilatation (FMD) in these subjects [37, 39]. Similar results were obtained in patients with previous myocardial infarction (MI) receiving 10 mg of resveratrol daily for 3 months [40]. However, the duration of these clinical trials was too short in order to assess the long-term consequences of the dietary intervention.

No clinical trials are available yet exploring the BP lowering effect of resveratrol in hypertensive patients.

### 2.3. Atherosclerosis and Dyslipidemia: Preclinical Studies

Resveratrol acts at the very early stages of atherosclerosis by increasing the hepatic uptake of low-density lipoprotein (LDL) through an AMPK independent mechanism and by reducing the expression of* intercellular adhesion molecule-1* (ICAM-1) and of vascular cell adhesion molecule-1 (VCAM-1) on endothelium [41, 42]. Additional in vitro studies demonstrated that resveratrol, likely via the phosphatidylinositol 3′-kinase (PI3K)/protein kinase B (PKB or Akt) pathway, blunts MCP-1 and chemokine receptor type 2 expression in monocytes [43, 44]. Also, it reduces foam cell formation by upregulating the expression of cholesterol transporters and by downregulating the uptake of oxidized LDL (Ox-LDL) [45]. The anti-inflammatory and antioxidant properties of resveratrol may be responsible for inhibition of LDL oxidation, of macrophage migration and transformation into foam cells, as well as of VSMCs migration and proliferation [46, 47].

Several in vivo studies have shown the hypocholesterolemic effect of a standard dose of resveratrol (20 mg/kg/day) [48, 49]. In the apolipoprotein (APO) E^−/−^ mice, resveratrol downregulated the hepatic 3-hydroxy 3-methylglutaryl coenzyme A (HMG-CoA) reductase enzyme, a key enzyme involved in cholesterol biosynthesis, thus reducing total and LDL cholesterol and increasing high-density lipoprotein (HDL) cholesterol [50]. In the high fat fed mice, resveratrol increased liver expression of cholesterol 7*α*-hydroxylase (CYP7A1) which led to increased bile acid synthesis and secretion, thus lowering the plasma level of total and LDL cholesterol [51].

#### 2.3.1. Atherosclerosis and Dyslipidemia: Clinical Studies

A meta-analysis evaluating the benefits of resveratrol supplementation on plasma lipids revealed no significant effect on any of the lipid parameters (e.g., total LDL and HD-cholesterol and triglycerides) independently of the dose, duration of the study, and cardiovascular risk of the considered population [52]. However, few single studies included in this meta-analysis reported that a relatively low dose of resveratrol treatment (250 mg per day for 3 months) led to a significant decrease of total cholesterol, total and ox-LDL, and ApoB levels in patients with type 2 diabetes mellitus (T2DM), coronary artery disease (CAD), hyperlipidemia, and other cardiovascular risk factors [53]. Similarly, total cholesterol and triglyceride levels were reduced by a very low dose of resveratrol (20 mg/per day for 2 months) in patients with stable angina [54].

### 2.4. Obesity and T2DM: Preclinical Studies

Resveratrol reduced lipid accumulation both in vivo and in vitro by inhibiting lipogenesis, increasing apoptosis, and promoting lipolysis [55–57]. In Sprague-Dawley rats the body fat-lowering effect of 30 mg resveratrol/kg body weight/day was mediated, at least, in part, by reduction in fatty acid uptake from circulating triacylglycerols, as well as by a de novo lipogenesis in adipose tissue [58]. In addition, it modulated insulin signaling pathway and improved insulin sensitivity in adipose and muscle tissue, as well as glucose uptake and insulin secretion [59, 60]. In human muscle cells derived from T2DM patients, resveratrol may improve glucose utilization and resistance to hyperglycemia by inhibiting phosphorylation of Insulin Receptor Substrate-1 [61].

In vivo, resveratrol restores vascular function through antioxidant, anti-inflammatory, and antiapoptotic properties, as it was observed treating rats with very low doses (0,75 mg/kg/three times a day) [62]. At a standard dose of 20 mg/kg/day, it improved cardiac function in both type 1 and type 2 DM [63–65].

#### 2.4.1. Obesity and T2DM: Clinical Studies

Resveratrol, at the standard dosage of 500 mg three times a day, improved insulin sensitivity in both obese and metabolic syndrome patients [66, 67]. However, other studies failed to confirm these findings [68, 69]. Anti-inflammatory effects of resveratrol were reported in several clinical studies performed in patients with high cardiovascular risk profile [54, 70]. Administration of resveratrol using different chemical formulae at several dosages was associated with decreased oxidative stress in patients with metabolic syndrome [71].

### 2.5. Ischemic Heart Disease: Preclinical Studies

Resveratrol protects against ischemic heart disease through multiple mechanisms. The mechanisms underlying the preconditioning effect of resveratrol (0,5 mg/kg/day) appear to be mainly mediated by NO and the antioxidant enzyme heme oxygenase-1 (HO-1) [72].

In vitro studies showed that resveratrol upregulated vascular endothelial growth factor (VEGF) expression in cardiomyocytes and in ECs through an increased oxidative-stress related proteins Thioredoxin-1 (Trx-1) and HO-1 expression [73]. It also protected cardiac tissue from cell death through multiple mechanisms including antiapoptotic effects and autophagy [74, 75].

Pretreatment of rats with resveratrol resulted in cardioprotection when the isolated heart was subjected to 30 min global ischemia followed by 2 hr reperfusion, or following permanent left anterior descending coronary artery (LAD) occlusion [76]. Resveratrol can potentiate regeneration of infarcted myocardium in a LAD occlusion rat model by stimulating neovascularization and cardiac stem cells [76, 77]. Interestingly, pretreatment with resveratrol largely restored the altered microRNAs expression in the ischemic heart [78].

The protective effects of resveratrol in the ischemic myocardium were confirmed in vivo [72, 79]. An interesting study conducted by Kanamori et al. suggested that only high dose (50 mg/kg/day) of resveratrol may be an effective treatment for ischemic heart failure (HF) by preventing necrotic area expansion and by improving cardiac function. Authors tested two doses of resveratrol (5 mg/kg and 50 mg/kg) demonstrating the dose-dependent effect of this compound [80].

#### 2.5.1. Ischemic Heart Disease: Clinical Studies

Few clinical trials investigated the effects of both standard and low doses of resveratrol in stable angina, acute coronary syndromes, and previous MI with positive results [40, 54, 70]. Additional studies suggested that resveratrol may be cardioprotective through increase of adiponectin and reduction of thrombogenic plasminogen activator inhibitor type 1 (PAI-1) [81, 82].

### 2.6. Cardiac Hypertrophy and Heart Failure: Preclinical Studies

Resveratrol was shown to prevent cardiac hypertrophy and dysfunction through reduction of oxidative stress, inhibition of hypertrophic gene expression, and increase of Ca^2+^ handling [83]. The antihypertrophic effect of resveratrol may be BP independent. For instance, low doses of resveratrol (2.5 mg/kg/day) prevented cardiac hypertrophy without reducing BP in SHR and Dahl-salt sensitive rats [84, 85]. The cardioprotective properties were demonstrated in several animal models, including pressure-overload, volume overload, SHR, doxorubicin-induced cardiotoxicity, myocarditis, MI, and ischemia-reperfusion (I/R) injury [15, 84, 86–90]. Recently, Sung et al. demonstrated that high doses of resveratrol (320 mg/kg/day) promote beneficial remodeling and improve both diastolic function and cardiac energy metabolism in a mice model of pressure-overload HF, thus increasing animal survival [91].

#### 2.6.1. Cardiac Hypertrophy and Heart Failure: Clinical Studies

In one study, performed in patients with HF of ischemic origin, treatment with resveratrol significantly improved diastolic function and induced a modest increase of systolic performance, despite the low dose administered (10 mg of resveratrol capsule/day) [40].

### 2.7. Cerebrovascular Disease: Preclinical Studies

The previously described beneficial vascular properties of resveratrol can also explain protection from ischemic stroke [92]. In vitro resveratrol promoted angiogenesis in cerebral ECs and prevented impairment of eNOS-dependent vasorelaxation of cerebral arterioles in diabetes [93, 94]. It also reduced infarct size in a rat model of focal cerebral ischemia and preserved blood brain barrier function by interfering with occludin and zonula occludens- (ZO-)1 tight junctions [92, 95, 96]. The stroke protective effects of resveratrol were also attributed to its specific neuroprotective properties [97, 98].

#### 2.7.1. Cerebrovascular Disease: Clinical Studies

There are no clinical studies investigating the protective effects of resveratrol in stroke patients. Interestingly, a single dose (250 mg) of* trans*-resveratrol increased cerebral blood flow during a mental stress (cognitive tasks) in healthy adult subjects [99].

### 2.8. Other Cardiovascular Diseases: Preclinical Studies

Resveratrol protected from doxorubicin-induced cardiotoxicity in a variety of animal models through the above discussed mechanisms [100–102]. However, there is scarce information on the cardioprotective effects of resveratrol in cancer patients treated with either doxorubicin or other cardiotoxic chemotherapeutic agents.

Few studies suggested an antiarrhythmic property. In fact, resveratrol caused a significant antiarrhythmic effect in three models of arrhythmia: aconitine-induced, ouabain-induced, and coronary ligation-induced arrhythmias [103]. Furthermore, chronic oral low-dose resveratrol treatment (5 mg/kg/day for 4 weeks starting one week before MI) significantly suppressed MI-induced ventricular tachycardia and ventricular fibrillation [104]. Recently, Baczko et al. designed and characterized a multifunctional resveratrol-derived small molecule, compound 1, targeting a number of key pathways involved in atrial fibrillation (AF), able to reduce the average and total AF duration in a model of inducible AF in conscious dogs [105].

## 3. *Brassica oleracea*

*Brassica oleracea* (BO) is a commonly used phytochemical. The species include broccoli, cauliflowers, Brussel sprouts, and kale. BO is highly enriched with bioactive molecules, whose effects on health have been partly explored [106–108]. It is known that the content of vitamin C varies significantly between the different subspecies of* Brassica*. These differences mainly depend on genotype and also on industrial storing, processing, and domestic cooking that reduce the final levels of available antioxidant compounds [109].

BO, in particular broccoli sprouts, is rich in glucosinolates: they are large molecules composed by a *β*-D-thioglucose group, a sulphonated oxime group and an amino-acidic side chain [110]. Sulforaphane is the active metabolite of glucoraphanin and is produced after hydrolyzation by myrosinase enzyme [111]. Cooking the vegetables partially denatures myrosinase; however, when glucoraphanin reaches the intestinal flora myrosinase-producing bacteria release the active metabolite that is then absorbed [112]. After absorption, sulforaphane is partly conjugated with glutathione in the liver, forming sulforaphane-glutathione. After reaching the kidneys, where it becomes sulforaphane-N-acetylcysteine, it is finally excreted in the urine [113]. Other active compounds of* Brassica* plants are anthocyanins, carotenoids, vitamin C, tocopherol, folic acid, and minerals. We will focus our discussion on sulforaphane and anthocyanins, as the main components of BO.

### 3.1. Molecular Targets and Properties

Molecular targets of BO include NF-*κ*B, nuclear factor-2 (Nrf2), mitogen-activated protein kinase (MAPK), c-Jun N-terminal kinase (JNK), Akt/PKB, and AMPK/SIRT-1/peroxisome proliferator-activated receptor-*α* (PPAR*α*)/uncoupling protein-2 (UCP2) [114–117]. By interacting with these molecular signaling pathways, BO plays antioxidant, anti-inflammatory, and antithrombotic effects.

### 3.2. Preclinical Studies

Broccoli sprouts exert several cardiovascular beneficial effects [106, 107].

With regard to glucosinolates, sulforaphane has been proven to be the most beneficial. In vitro, it induced expression of detoxification enzymes, the so called “ARE” targets (Antioxidant Response Elements: nicotinamide adenine dinucleotide (NADH) quinone reductase, HO-1, and glutathione transferase), and several nuclear factors, such as Nrf2, involved in ROS elimination and xenobiotic excretion [114]. Sulforaphane suppressed the expression of MAPK p38 in ECs through activation of Nrf2, thus leading to reduction of VCAM-1 synthesis [118]. By reducing ROS, sulforaphane lowered ox-LDL level in blood [119]. In a study conducted in rats fed for 14 weeks with 200 mg/day of dried broccoli sprouts, a significant increase in glutathione content was observed along with increased glutathione reductase and peroxidase (GPx) activities in both heart and kidneys [120]. Interestingly, administration of broccoli sprouts in pregnant female stroke prone-SHR (SHRSP) decreased oxidative stress and BP levels, compared to females fed with control diet. Furthermore, offspring of females maintained on broccoli diet during pregnancy had also lower BP and tissue inflammation in adulthood, regardless of diet [121].

Anthocyanins are known to promote optimal platelet function and antithrombotic effects [122]. These compounds can act on different types of cells involved in atherosclerosis development. In fact, they exert a protective effect toward TNF-*α* induced MCP-1 secretion in primary human ECs [123]. Anthocyanins prevented the expression of VEGF stimulated by platelet derived growth factor (PDGF) AB in VSMCs and by MAPK p38 and c-JNK inhibition [115]. Moreover, anthocyanins extract induced endothelium-dependent relaxation in porcine coronary arteries [124]. Increased cardiac glutathione concentrations in rats receiving long-term administration of anthocyanins contributed to the antioxidant effects [125]. The protective effect on heart also depends on reduction of hypertrophy-associated increased phosphorylation of PKC and on activation of Akt/PKB [116]. Moreover, anthocyanins prevented CD40-activated proinflammatory signaling in ECs by regulating cholesterol distribution [126]. They also inhibit the activation of NF-*κ*B and lipopolysaccharides induced NO biosynthesis in macrophages [127].

Broccoli sprouts protect from myocardial oxidative damage and cell death in ischemia/reperfusion (I/R) rat models. In particular, anthocyanins decreased the extent of cell death in cultured cardiomyocytes and reduced infarct size by inhibiting signal transducer and activator of transcription 1 (STAT1) stimulation [106, 128]. Notably, BO improved diabetic nephropathy in rats [107] and prevented renal damage in salt-loaded SHRSP, independently from SBP, through AMPK/SIRT1/PPAR*α*/UCP2 axis activation [117]. In fact, selective inhibition of PPAR*α* antagonized the nephroprotective effects of BO sprouts, consistently with previous evidence on the role of cyanidin as PPAR*α* agonist [129].

### 3.3. Clinical Studies

The beneficial effects in humans were enhanced when broccoli supplements were combined with fresh broccoli sprouts administration in healthy subjects who consumed either 68 gr of broccoli sprouts or 6 Brocco-Max pills (about 3 gr of freeze-dried broccoli sprouts in 6 pills) for 7 days [130, 131]. In a small clinical trial conducted in 6 men and 6 women, all smokers, eating 100 gr of broccoli sprouts daily for 7 days, a significant reduction of both total and LDL cholesterol along with urinary 8-isoprostanes and other markers of oxidative stress was observed [119]. The administration of 150 mL/day kale juice for 12 weeks in 32 men with hypercholesterolemia significantly reduced plasma LDL cholesterol and increased both HDL cholesterol and GPx activity, thus lowering CAD risk [108]. In addition, broccoli sprouts supplement could play favorable effects on lipid profiles and OX-LDL/LDL cholesterol ratio in T2DM [132]. Recently, anthocyanins intake (8,4–23,6 mg/day) was shown to associate with lower arterial stiffness and central BP in women [133].

Results from human trials are controversial. In fact, Curtis et al. showed no effect on markers of CVDs (including inflammatory biomarkers, platelet reactivity, lipids, and glucose), on liver and kidney function, as well as on anthropometric parameters, BP, and heart rate, following 12-week intervention with 500 mg/day cyanidin in postmenopausal women [134].

## 4. Curcumin

Curcumin (diferuloylmethane) is a naturally occurring phenolic compound isolated as a yellow pigment from spice turmeric (Curcuma Longa). This compound has received attention due to its various biological and pharmacological activities. Its therapeutic effects have been extensively investigated, particularly in the treatment of cancer and inflammatory diseases [135].

There is growing evidence that curcumin has a potential role in protection from several CVDs [135, 136].

### 4.1. Molecular Targets and Properties

Curcumin interacts with different molecular targets, such as Janus Kinase 2 (JAK2)/STAT3, AMPK/UCP2, Akt/Nrf2, ERK, MAPK p38, JNK, ICAM-1, MCP-1, and IL-8 [137–141]. As a consequence, it exerts anti-inflammatory, antiplatelet, and antioxidant properties [141–144]. Concerning the latter, a single dose of 15 mg/kg of curcumin appears to decrease levels of xanthine oxidase, superoxide anion, lipid peroxides, and myeloperoxidase and to increase levels of SOD, catalase, GPx, and glutathione-S-transferase (GST) [145]. Moreover, this phytochemical reduces level of eNOS and iNOS through the activation of NF-*κ*B and protein-1 (AP-1) [146]. Curcumin is also a potent inducer of HO-1 in ECs through activation of ARE in several cardiovascular cells exposed to curcumin 5–15 *μ*M [147]. Moreover, curcumin appears to attenuate mitochondrial alterations and respiratory cellular dysfunction [148].

### 4.2. Preclinical Studies

Curcumin plays a protective role on endothelium by inducing HO-1 in bovine aortic ECs [147]. It exerts antiproliferative and antiapoptotic effects on VSMCs, exposed to 1–25 *μ*M of curcumin, thus attenuating carotid artery neointima formation [149–151]. It plays a relevant role on calcium homeostasis in both skeletal muscle and cardiac sarcoplasmic reticulum [152].

The role of curcumin in CVDs has been investigated in several animal models. For instance, 1,66 mg curcumin/kg showed a hypolipidemic effect and protection from aortic fatty streak development [153, 154]. The cardioprotective role of curcumin was shown in myocardial ischemia rat models [145, 155]. In I/R models, curcumin reduced collagen synthesis and fibrosis and significantly improved left ventricular end-diastolic volume, stroke volume, and ejection fraction [156]. In addition, it reduced MI size and depressed lactate dehydrogenase release in the coronary blood flow through activation of JAK2/STAT3 [137]. These beneficial effects could be related to a decrease of proinflammatory cytokines and of cardiomyocyte apoptosis [157].

In two different HF models, 50 mg curcumin/kg/day ameliorated systolic function and prevented myocardial hypertrophy by inhibiting p300-HAT (histone acetyltransferases) [158]. Additionally, a larger amount of curcumin (200 mg/kg/day) showed a protective role in adriamycin-induced cardiac damage [159] and it also prevented cardiovascular complications in diabetes [146]. In fact, it reduced high glucose-induced overexpression of inflammatory cytokines in macrophages [144]. A beneficial role toward myocardial injury was reported in renal I/R injury rat models [160].

Finally, a standard dose of curcumin (25–50 mg/kg/day) protected against cerebral ischemic insult [161], as well as aging-related cerebrovascular dysfunction via AMPK/UCP2 pathway. It protected neurons against ischemic injury through Akt/Nrf2 pathway [138, 139]. In different stroke models curcumin not only decreased oxidative stress but also attenuated reperfusion injury by preventing neutrophil adhesion to the cerebrovascular microcirculation [162, 163].

### 4.3. Clinical Studies

Controversial results exist with regard to the effect of curcumin on plasma lipids in healthy subjects. In fact, in healthy volunteers, a dosage of 500 mg curcumin/day decreased both serum lipid peroxides and total cholesterol and increased HDL cholesterol [164]. Hypolipidemic effects were also observed in patients affected by atherosclerosis, acute coronary syndrome, and T2DM. Moreover, the effect of curcumin administration on lipid profile was evaluated in acute coronary syndrome (ACS) patients at escalating doses (low dose, 3 times 15 mg/day; moderate dose, 3 times 30 mg/day; high dose, 3 times 60 mg/day). Unexpectedly, this study showed that the low dose of curcumin was associated with higher reduction of total, HDL and LDL cholesterol levels [165, 166]. On the other hand, a meta-analysis failed to show protective effects of curcumin on both cholesterol and triglycerides in a heterogeneous population [167]. Curcumin administration and aerobic exercise training increased FMD in postmenopausal women [168]. Interestingly, curcumin may improve the blood compatibility of rapamycin-eluting stents through its antiplatelet properties [169].

## 5. Berberine

Berberine (BBR), an alkaloid isolated from* Hydrastis canadensis*, the Chinese herb Huanglian, and many other plants, such as the Berberidaceae and Ranunculaceae families, has a long history in traditional Chinese medicine. BBR is present in roots, rhizomes, and stem bulk of the plants. Various pharmacological actions, including antibiotic, immunostimulant, antitumor, and antimotility properties have been described for BBR [170, 171].

Recent studies have indicated that BBR may be also effective in treating chronic, multifactorial diseases, including diabetes, hyperlipidemia, heart diseases, cancer, neurological disorders, and inflammatory diseases [172, 173].

### 5.1. Molecular Targets and Properties

Molecular mechanisms mediating antioxidant effects appear to be mainly related to upregulation of both SOD and UCP2 and to downregulation of NADPH oxidase expression [174, 175] with particular regard to NADPH oxidase 2/4 subunits [175]. BBR administration activates Nrf2 pathway, which is crucial for antioxidant and anti-inflammatory activities [176]. BBR could suppress inflammation by blocking the MAPK pathways in a AMPK-dependent manner, by inhibiting the NF-*κ*B signaling pathway and the Rho GTPase pathway and by attenuating transcription activity of AP-1, which is possibly mediated by PPAR*α* activation [177–179].

### 5.2. Preclinical Evidences

In vitro studies demonstrated the role of BBR in counteracting endothelial progenitor cells (EPCs) dysfunction. In fact, BBR improved the proliferative ability of EPCs impaired by TNF-*α* via activation of PI3K/Akt/eNOS signaling pathway [180]. Moreover, BBR induced endothelium-dependent vasorelaxation and enhanced endothelium-independent VSMC dilatation through a partial reduction of oxidative stress [181].

In VSMCs, isolated from thoracic aorta of Sprague-Dawley rats, BBR inhibited Ang II- and heparin binding epidermal growth factor- (HB-EGF-) induced VSMC proliferation and migration. In vivo results showed a reduction of neointima formation after balloon injury, thus lowering risk of restenosis [182]. Zimetti et al. demonstrated a double protective effect of BBR on cholesterol homeostasis underlying foam cells formation and on the inflammatory phenotype in mouse and human macrophages [183].

BBR affected glucose metabolism by increasing insulin secretion, stimulating glycolysis, suppressing adipogenesis, and increasing glucokinase activity and both glucose transporter-4 (GLUT-4) and glucagon-like peptide (GLP-1) levels in glucose-consuming tissues [184].

Furthermore, BBR was shown to have lipid-lowering properties in animals as well as in hyperlipidemic patients through mechanisms different from those of statins, involving activation of ERK pathway and increase of LDLR expression on the hepatocytes surface [185]. Interestingly, contrasting results were reported with regard to modulation of the gene encoding proprotein convertase subtilisin kexin 9 (PCSK9), a natural inhibitor of LDLR. In HepG2 cells 20 *μ*M BBR downregulated the transcription of the gene [186], whereas 400 mg BBR/kg/day significantly reduced body weight and improved lipid profile by increasing the PCSK9 expression levels through Sterol Regulatory Element-Binding Proteins activation in the high fat diet (HFD) rat model [187].

BBR, at the dosage of 100 mg/kg/day, plays positive inotropic, antiarrhythmic, and vasodilator properties related to the cardiovascular system [188, 189]. The antiarrhythmic effects are due, at least in part, to preferential blockade of the components of the delayed rectifying potassium current, I(Kr), and I(Ks) and to increased effective refractory period of Purkinje fibers [190, 191].

The beneficial effects of BBR were demonstrated in several animal models such as SHR, HFD rats, pressure-overload HF, and myocardial ischemia [187, 192–194]. Notably, 50 Sprague-Dawley rats were treated with BBR (30 or 60 mg/kg) demonstrating that BBR had cardioprotective effects against acute ischemic myocardial injury in a dose-dependent manner [194]. BBR counteracted several pathological features of hypertension, including suppression of endoplasmic reticulum stress, inhibition of ROS accumulation, and attenuation of endothelium-dependent contractions in SHR [195]. The antihypertensive effect of BBR derivative 6-protoberberine (PTB-6) was shown in conscious SHR and Wistar-Kyoto (WKY) rats, and it was mediated by reduced SNS activity through a negative inotropic and chronotropic effect [192].

A recent in vivo study reported that BBR can prevent cardiac hypertrophy and attenuate cardiomyocyte apoptosis in the transverse aortic contraction treated rat model [193].

In a rat model of MI, BBR administration significantly enhanced autophagic activity, attenuated adverse left ventricular remodelling, and preserved left ventricular systolic function. Interestingly, low-dose BBR (10 mg/kg per day) was associated with greater improvement in cardiac function compared with high-dose BBR (50 mg/kg per day) [196]. In diabetic rat models, BBR protected the heart against I/R injury, improved cardiac function, and reduced myocardial apoptosis via activation of AMPK and PI3K/Akt and eNOS signaling [197]. In addition, cardioprotective effects of BBR in myocardial ischemia are due to its antioxidant and anti-inflammatory properties [194].

Chronic administration of BBR significantly reduced oxidative stress and vascular inflammation and suppressed atherogenesis in ApoE^−/−^ mice by AMPK-dependent UCP2 expression [174].

In a middle cerebral artery occlusion (MCAO) model, BBR improved neurological outcome and reduced I/R-induced cerebral infarction 48 hrs after MCAO. The protective effect of BBR was confirmed in vitro [198].

### 5.3. Clinical Evidences

BBR has shown good safety results in human studies [199]. A randomized clinical trial tested its effects in 156 patients with chronic congestive HF. The BBR-treated group (1,2–2 gr/day) showed significantly greater increases in left ventricular ejection fraction and exercise capacity, significant improvements on the dyspnea-fatigue index, and decreased rates of ventricular premature complexes and long-term mortality [200].

Treatment of 100 arrhythmic patients with BBR resulted in a >89% reduction in premature beating in the majority of patients and >50% reduction in the remaining patients [201]. These results were independently reproduced [202]. A recent meta-analysis, including 11 randomized controlled studies (874 Chinese participants affected by hyperlipidemia, T2DM, or both diseases), has shown a significant reduction in total cholesterol, triglycerides, and LDL cholesterol levels and a small but significant increase in HDL cholesterol [203].

In T2DM patients, high-dose BBR administration (100–200 mg/kg/day) was associated with a significant reduction in glycated hemoglobin, fasting plasma insulin, postprandial glucose, and fasting plasma glucose [204].

BBR beneficial effects were also observed in hypercholesterolemic European patients [205].

A recent meta-analysis emphasized the role of BBR in the treatment of hypertension. In fact, BBR associated with lifestyle intervention tended to lower the level of BP more than lifestyle intervention alone or than placebo [206].

## 6. Conclusions

Preclinical studies revealed several beneficial cardiovascular effects of resveratrol,* Brassica oleracea*, curcumin, and berberine. The benefits appeared to be mainly dependent on antioxidant, anti-inflammatory, and antithrombotic properties. In fact, the excellent results of both in vitro and in vivo studies induced researchers and clinicians to test the effects of phytochemicals in humans. However, evidences obtained from the few available clinical trials on the protective effects of these compounds in several CVDs are still controversial. A main limitation of current clinical studies relies on their heterogeneity and on small samples size. Furthermore, based on the literature discussed in the present paper, some confusion arises about the precise dose of each compound exerting more pronounced beneficial effects. In particular, whereas the use of a very high dose is associated with the most protective effects for few phytochemicals, the lowest dose turns out to be the most effective for other compounds. This phenomenon appears to be related to different animal models as well as to the specific disease under consideration. Therefore, there is a need for additional larger and well controlled human studies.

Altogether, the lack of a clear beneficial role in humans, the wide variety of in vitro, ex vivo, and in vivo experimental evidences that are summarized in Tables 1 and 2, suggests that resveratrol,* Brassica oleracea*, curcumin, and berberine may reveal very useful preventive and/or therapeutic tools for the treatment of CVDs, as a valid support to medical therapies.

## Figures and Tables

**Table 1:** Preclinical effects of vegetable compounds.

Vegetable	Preclinical effects	Reference
Resveratrol	(i) Upregulates the **antioxidant system** and reduces **ROS** production	[14, 15]
	(ii) Inhibits **vascular inflammation** and prevents **platelet activation**	[16–22]
	(iii) Lowers **BP **in animals with either diabetes or metabolic syndrome	[33]
	(iv) Inhibits very early stages of **atherosclerosis**	[41, 42]
	(v) Reduces **lipid accumulation** by inhibiting lipogenesis, increasing apoptosis, and promoting lipolysis	[55–57]
	(vi) Protects against **ischemic heart disease**	[72]
	(vii) Protects cardiac tissue from **cell death **though apoptosis and autophagy	[74, 75]
	(viii) Potentiates **regeneration of infarcted myocardium**	[76, 77]
	(ix) Prevents cardiac hypertrophy and dysfunction	[83]
	(x) Promotes **angiogenesis** in cerebral ECs and prevents impairment of eNOS-dependent **vasorelaxation** of cerebral arterioles	[93, 94]
	(xi) Protects from doxorubicin-induced **cardiotoxicity**	[100–102]
	(xii) **Antiarrhythmic effects**	[103]

*Brassica oleracea *	(i) Induces expression of **detoxification enzymes** (ARE targets)	[114]
	(ii) Lowers **ox-LDL** blood levels	[119]
	(iii) Decreases **oxidative stress and BP levels** in pregnant female SHRSP	[121]
	(iv) Promotes **optimal platelet function** and **antithrombotic** effects	[122]
	(v) Acts on different types of cells involved in **atherosclerosis** development	[123]
	(vi) Regulates **cholesterol** distribution	[126]
	(vii) Protects from **myocardial oxidative damage and cell death** in ischemia-reperfusion rat models	[106, 128]
	(viii) **Nephroprotective** effects	[129]

Curcumin	(i) Protective role on **endothelium** by inducing HO-1	[147]
	(ii) **Antiproliferative** and **antiapoptotic** effects on VSMCs, attenuating **neointima formation**	[149–151]
	(iii) **Hypolipidemic** effect and protection from **aortic fatty streak** development	[153, 154]
	(iv) Reduces collagen synthesis and **fibrosis and** improves **left ventricular end-diastolic volume, stroke volume, and ejection fraction**	[156]
	(v) Reduces **MI** size	[137]
	(vi) Protects from **adriamycin-induced cardiotoxicity**	[159]
	(vii) Protects from **cerebral ischemic insult**	[138, 139]

Berberine	(i) Improves the **proliferative ability of EPCs**	[180]
	(ii) Induces **endothelial-dependent vasorelaxation** and enhances **endothelium-independent VSMC dilatation**	[181]
	(iii) Inhibits VSMC proliferation and migration and reduces **neointima formation**	[182]
	(iv) **Lipid-lowering **properties	[185]
	(v) **Positive inotropic, antiarrhythmic, and vasodilator **properties	[188, 189]
	(vi) **Antihypertensive effects** in SHR	[195]
	(vii) Prevents **cardiac hypertrophy** and attenuates cardiomyocyte apoptosis	[193]
	(viii) Attenuates **adverse left ventricular remodeling and preserves left ventricular systolic function** in rat model of MI	[196]

ROS: reactive oxygen species; BP: blood pressure; eNOS: endothelial nitric oxide sinthase; ARE: Antioxidant Response Elements; ox-LDL: oxidized low-density lipoprotein; SHRSP: stroke prone spontaneously hypertensive rats; HO-1: heme oxygenase-1; ECs: endothelial cells; VSMC: vascular smooth muscular cells; MI: myocardial infarction; EPCs: endothelial progenitor cells.

**Table 2:** Clinical effects of vegetable compounds.

Vegetable	Clinical effects	Reference
Resveratrol	(i) Decreases **SBP** without affecting DBP	[37]
	(ii) Enhances **Ach-mediated vasorelaxation** in hypertensive and dyslipidemic pts	[38]
	(iii) Improves **FMD** in pts with either metabolic syndrome or previous MI	[39, 40]
	(iv) Decreases **total cholesteroland total **and **ox-LDL, triglycerides**, and** ApoB** levels in pts with T2DM, CAD, hyperlipidemia, and other CV risk factors	[53, 54]
	(v) Improves **insulin sensitivity** in both obese and metabolic syndrome pts	[66, 67]
	(vi) Improves significantly **diastolic function** and modestly **systolic function **in pts with previous MI	[40]

*Brassica oleracea *	(i) Reduces **total, LDL cholesterol **and **markers of oxidative stress** in smokers and hypercholesterolemic pts	[108, 119]
	(ii) Improves lipid profiles and **ox-LDL/LDL cholesterol ratio** in T2DM pts	[132]
	(iii) Lowers **arterial stiffness** and **central BP** in women	[133]

Curcumin	(i) Decreases both **total and LDL cholesterol** and increases **HDL cholesterol **in healthy subjects and in ACS pts	[164, 165]
	(ii) Increases **FMD** in postmenopausal women	[168]

Berberine	(i) Increases both **LVEF** and **exercise capacity** and decreases rates of **ventricular premature complexes** and **long-term mortality** in HF pts	[199]
	(ii) Reduces **total cholesterol, triglycerides, and LDL cholesterol** levels and modestly increases **HDL cholesterol** in hyperlipidemic and T2DM pts	[203, 205]
	(iii) Reduces **glycated hemoglobin, fasting plasma insulin, postprandial glucose, and fasting plasma glucose** in T2DM pts	[204]
	(iv) Lowers **SBP** and **DBP** in hypertensive, T2DM, and hyperlipidemic pts	[206]

SBP: systolic blood pressure; DBP: diastolic blood pressure; Ach: Acetylcholine; FMD: flow-mediated dilation; MI: myocardial infarction; ox-LDL: oxidized low-density lipoprotein; HDL: high-density lipoprotein; Apo: apolipoprotein; T2DM: type 2 diabetes mellitus; CAD: coronary artery disease; CV: cardiovascular; ACS: acute coronary syndrome; LVEF: left ventricular ejection fraction; HF: heart failure; pts: patients.

## References

[1] Mozaffarian D., Benjamin E. J., Go A. S. (2015). Executive summary: heart disease and stroke statistics-2015 update: a report from the American Heart Association. *Circulation*.

[2] Qin Y., Shu F., Zeng Y. (2014). Daidzein supplementation decreases serum triglyceride and uric acid concentrations in hypercholesterolemic adults with the effect on triglycerides being greater in those with the GA compared with the GG genotype of ESR-*β* RsaI. *Journal of Nutrition*.

[3] Chan E., Wong C. Y.-K., Wan C.-W. (2010). Evaluation of anti-oxidant capacity of root of *Scutellaria baicalensis* Georgi, in comparison with roots of *Polygonum multiflorum* Thunb and *Panax ginseng* CA Meyer. *The American Journal of Chinese Medicine*.

[4] Burns J., Yokota T., Ashihara H., Lean M. E. J., Crozier A. (2002). Plant foods and herbal sources of resveratrol. *Journal of Agricultural and Food Chemistry*.

[5] Mikulski D., Górniak R., Molski M. (2010). A theoretical study of the structure-radical scavenging activity of trans-resveratrol analogues and cis-resveratrol in gas phase and water environment. *European Journal of Medicinal Chemistry*.

[6] Das D. K., Mukherjee S., Ray D. (2010). Resveratrol and red wine, healthy heart and longevity. *Heart Failure Reviews*.

[7] Renaud S., de Lorgeril M. (1992). Wine, alcohol, platelets, and the French paradox for coronary heart disease. *The Lancet*.

[8] Cottart C.-H., Nivet-Antoine V., Beaudeux J.-L. (2014). Review of recent data on the metabolism, biological effects, and toxicity of resveratrol in humans. *Molecular Nutrition and Food Research*.

[9] Yun H., Park S., Kim M.-J. (2014). AMP-activated protein kinase mediates the antioxidant effects of resveratrol through regulation of the transcription factor FoxO1. *FEBS Journal*.

[10] Saud S. M., Li W., Morris N. L. (2014). Resveratrol prevents tumorigenesis in mouse model of Kras activated sporadic colorectal cancer by suppressing oncogenic Kras expression. *Carcinogenesis*.

[11] Cao X., Luo T., Luo X., Tang Z. (2014). Resveratrol prevents AngII-induced hypertension via AMPK activation and RhoA/ROCK suppression in mice. *Hypertension Research*.

[12] Zarzuelo M. J., López-Sepúlveda R., Sánchez M. (2013). SIRT1 inhibits NADPH oxidase activation and protects endothelial function in the rat aorta: implications for vascular aging. *Biochemical Pharmacology*.

[13] Pacholec M., Bleasdale J. E., Chrunyk B. (2010). SRT1720, SRT2183, SRT1460, and resveratrol are not direct activators of SIRT1. *The Journal of Biological Chemistry*.

[14] Spanier G., Xu H., Xia N. (2009). Resveratrol reduces endothelial oxidative stress by modulating the gene expression of superoxide dismutase 1 (SOD1), glutathione peroxidase 1 (GPx1) and NADPH oxidase subunit (Nox4). *Journal of Physiology and Pharmacology*.

[15] Tanno M., Kuno A., Yano T. (2010). Induction of manganese superoxide dismutase by nuclear translocation and activation of SIRT1 promotes cell survival in chronic heart failure. *The Journal of Biological Chemistry*.

[16] Moreno J. J. (2000). Resveratrol modulates arachidonic acid release, prostaglandin synthesis, and 3T6 fibroblast growth. *Journal of Pharmacology and Experimental Therapeutics*.

[17] Kundu J. K., Shin Y. K., Kim S. H., Surh Y.-J. (2006). Resveratrol inhibits phorbol ester-induced expression of COX-2 and activation of NF-*κ*B in mouse skin by blocking I*κ*B kinase activity. *Carcinogenesis*.

[18] Yang L., Zhang J., Yan C. (2012). SIRT1 regulates CD40 expression induced by TNF-*α* via NF-*κ*B pathway in endothelial cells. *Cellular Physiology and Biochemistry*.

[19] Cullen J. P., Morrow D., Jin Y. (2007). Resveratrol, a polyphenolic phytostilbene, inhibits endothelial monocyte chemotactic protein-1 synthesis and secretion. *Journal of Vascular Research*.

[20] Wu C. C., Wu C. I., Wang W. Y., Wu Y. C. (2007). Low concentrations of resveratrol potentiate the antiplatelet effect of prostaglandins. *Planta Medica*.

[21] Lin K. H., Hsiao G., Shih C. M., Chou D. S., Sheu J. R. (2009). Mechanisms of resveratrol-induced platelet apoptosis. *Cardiovascular Research*.

[22] Shen M. Y., Hsiao G., Liu C. L. (2007). Inhibitory mechanisms of resveratrol in platelet activation: pivotal roles of p38 MAPK and NO/cyclic GMP. *British Journal of Haematology*.

[23] Olas B., Wachowicz B., Holmsen H., Fukami M. H. (2005). Resveratrol inhibits polyphosphoinositide metabolism in activated platelets. *Biochimica et Biophysica Acta—Biomembranes*.

[24] Rush J. W. E., Quadrilatero J., Levy A. S., Ford R. J. (2007). Chronic resveratrol enhances endothelium-dependent relaxation but does not alter eNOS levels in aorta of spontaneously hypertensive rats. *Experimental Biology and Medicine*.

[25] Novakovic A., Gojkovic-Bukarica L., Peric M. (2006). The mechanism of endothelium-independent relaxation induced by the wine polyphenol resveratrol in human internal mammary artery. *Journal of Pharmacological Sciences*.

[26] Zou J., Wang Z., Huang Y., Cao K., Wu J. (2003). Effect of red wine and wine polyphenol resveratrol on endothelial function in hypercholesterolemic rabbits. *International Journal of Molecular Medicine*.

[27] Liu J.-C., Chen J.-J., Chan P., Cheng C.-F., Cheng T.-H. (2003). Inhibition of cyclic strain-induced endothelin-1 gene expression by resveratrol. *Hypertension*.

[28] Ma H.-J., Cao Y.-K., Liu Y.-X., Wang R., Wu Y.-M. (2008). Microinjection of resveratrol into rostral ventrolateral medulla decreases sympathetic vasomotor tone through nitric oxide and intracellular Ca^2+^ in anesthetized male rats. *Acta Pharmacologica Sinica*.

[29] Bhatt S. R., Lokhandwala M. F., Banday A. A. (2011). Resveratrol prevents endothelial nitric oxide synthase uncoupling and attenuates development of hypertension in spontaneously hypertensive rats. *European Journal of Pharmacology*.

[30] Toklu H. Z., Şehirli Ö., Erşahin M. (2010). Resveratrol improves cardiovascular function and reduces oxidative organ damage in the renal, cardiovascular and cerebral tissues of two-kidney, one-clip hypertensive rats. *Journal of Pharmacy and Pharmacology*.

[31] Liu Z., Song Y., Zhang X. (2005). Effects of trans-resveratrol on hypertension-induced cardiac hypertrophy using the partially nephrectomized rat model. *Clinical and Experimental Pharmacology and Physiology*.

[32] Chan V., Fenning A., Iyer A., Hoey A., Brown L. (2011). Resveratrol improves cardiovascular function in DOCA-salt hypertensive rats. *Current Pharmaceutical Biotechnology*.

[33] Aubin M.-C., Lajoie C., Clément R., Gosselin H., Calderone A., Perrault L. P. (2008). Female rats fed a high-fat diet were associated with vascular dysfunction and cardiac fibrosis in the absence of overt obesity and hyperlipidemia: therapeutic potential of resveratrol. *Journal of Pharmacology and Experimental Therapeutics*.

[34] Förstermann U., Li H. (2011). Therapeutic effect of enhancing endothelial nitric oxide synthase (eNOS) expression and preventing eNOS uncoupling. *British Journal of Pharmacology*.

[35] Mattagajasingh I., Kim C.-S., Naqvi A. (2007). SIRT1 promotes endothelium-dependent vascular relaxation by activating endothelial nitric oxide synthase. *Proceedings of the National Academy of Sciences of the United States of America*.

[36] Li H., Förstermann U. (2000). Nitric oxide in the pathogenesis of vascular disease. *Journal of Pathology*.

[37] Liu Y., Ma W., Zhang P., He S., Huang D. (2015). Effect of resveratrol on blood pressure: a meta-analysis of randomized controlled trials. *Clinical Nutrition*.

[38] Carrizzo A., Puca A., Damato A. (2013). Resveratrol improves vascular function in patients with hypertension and dyslipidemia by modulating NO metabolism. *Hypertension*.

[39] Wong R. H. X., Berry N. M., Coates A. M. (2013). Chronic resveratrol consumption improves brachial flow-mediated dilatation in healthy obese adults. *Journal of Hypertension*.

[40] Magyar K., Halmosi R., Palfi A. (2012). Cardioprotection by resveratrol: a human clinical trial in patients with stable coronary artery disease. *Clinical Hemorheology and Microcirculation*.

[41] Yashiro T., Nanmoku M., Shimizu M., Inoue J., Sato R. (2012). Resveratrol increases the expression and activity of the low density lipoprotein receptor in hepatocytes by the proteolytic activation of the sterol regulatory element-binding proteins. *Atherosclerosis*.

[42] Xiao J., Song J., Hodara V. (2013). Protective effects of resveratrol on TNF-*α*-induced endothelial cytotoxicity in baboon femoral arterial endothelial cells. *Journal of Diabetes Research*.

[43] Park D.-W., Baek K., Kim J.-R. (2009). Resveratrol inhibits foam cell formation via NADPH oxidase 1-mediated reactive oxygen species and monocyte chemotactic protein-1. *Experimental and Molecular Medicine*.

[44] Cullen J. P., Morrow D., Jin Y. (2007). Resveratrol inhibits expression and binding activity of the monocyte chemotactic protein-1 receptor, CCR2, on THP-1 monocytes. *Atherosclerosis*.

[45] Voloshyna I., Hai O., Littlefield M. J., Carsons S., Reiss A. B. (2013). Resveratrol mediates anti-atherogenic effects on cholesterol flux in human macrophages and endothelium via PPARgamma and adenosine. *European Journal of Pharmacology*.

[46] Csiszar A., Sosnowska D., Wang M., Lakatta E. G., Sonntag W. E., Ungvari Z. (2012). Age-associated proinflammatory secretory phenotype in vascular smooth muscle cells from the non-human primate macaca mulatta: reversal by resveratrol treatment. *Journals of Gerontology—Series A: Biological Sciences and Medical Sciences*.

[47] Li L., Gao P., Zhang H. (2011). SIRT1 inhibits angiotensin II-induced vascular smooth muscle cell hypertrophy. *Acta Biochimica et Biophysica Sinica*.

[48] Göçmen A. Y., Burgucu D., Gümüşlü S. (2011). Effect of resveratrol on platelet activation in hypercholesterolemic rats: CD40-CD40l system as a potential target. *Applied Physiology, Nutrition and Metabolism*.

[49] Wang Z., Zou J., Cao K., Hsieh T.-C., Huang Y., Wu J. M. (2005). Dealcoholized red wine containing known amounts of resveratrol suppresses atherosclerosis in hypercholesterolemic rabbits without affecting plasma lipid levels. *International Journal of Molecular Medicine*.

[50] Do G.-M., Kwon E.-Y., Kim H.-J. (2008). Long-term effects of resveratrol supplementation on suppression of atherogenic lesion formation and cholesterol synthesis in apo E-deficient mice. *Biochemical and Biophysical Research Communications*.

[51] Chen Q., Wang E., Ma L., Zhai P. (2012). Dietary resveratrol increases the expression of hepatic 7-hydroxylase and ameliorates hypercholesterolemia in high-fat fed C57BL/6J mice. *Lipids in Health and Disease*.

[52] Sahebkar A. (2013). Effects of resveratrol supplementation on plasma lipids: a systematic review and meta-analysis of randomized controlled trials. *Nutrition Reviews*.

[53] Bhatt J. K., Thomas S., Nanjan M. J. (2012). Resveratrol supplementation improves glycemic control in type 2 diabetes mellitus. *Nutrition Research*.

[54] Militaru C., Donoiu I., Craciun A., Scorei I. D., Bulearca A. M., Scorei R. I. (2013). Oral resveratrol and calcium fructoborate supplementation in subjects with stable angina pectoris: effects on lipid profiles, inflammation markers, and quality of life. *Nutrition*.

[55] Chen S., Li Z., Li W., Shan Z., Zhu W. (2011). Resveratrol inhibits cell differentiation in 3T3-L1 adipocytes via activation of AMPK. *Canadian Journal of Physiology & Pharmacology*.

[56] Mader I., Wabitsch M., Debatin K.-M., Fischer-Posovszky P., Fulda S. (2010). Identification of a novel proapoptotic function of resveratrol in fat cells: SIRT1-independent sensitization to TRAIL-induced apoptosis. *The FASEB Journal*.

[57] Lasa A., Schweiger M., Kotzbeck P. (2012). Resveratrol regulates lipolysis via adipose triglyceride lipase. *Journal of Nutritional Biochemistry*.

[58] Alberdi G., Rodríguez V. M., Miranda J. (2011). Changes in white adipose tissue metabolism induced by resveratrol in rats. *Nutrition & Metabolism*.

[59] Koren S., Fantus I. G. (2007). Inhibition of the protein tyrosine phosphatase PTP1B: potential therapy for obesity, insulin resistance and type-2 diabetes mellitus. *Best Practice and Research in Clinical Endocrinology and Metabolism*.

[60] Grundy S. M., Benjamin I. J., Burke G. L. (1999). Diabetes and cardiovascular disease: a statement for healthcare professionals from the American Heart Association. *Circulation*.

[61] Frojdo S., Durand C., Molin L. (2011). Phosphoinositide 3-kinase as a novel functional target for the regulation of the insulin signaling pathway by SIRT1. *Molecular and Cellular Endocrinology*.

[62] Jing Y.-H., Chen K.-H., Yang S.-H., Kuo P.-C., Chen J.-K. (2010). Resveratrol ameliorates vasculopathy in STZ-induced diabetic rats: role of AGE-RAGE signalling. *Diabetes/Metabolism Research and Reviews*.

[63] Thirunavukkarasu M., Penumathsa S. V., Koneru S. (2007). Resveratrol alleviates cardiac dysfunction in streptozotocin-induced diabetes: role of nitric oxide, thioredoxin, and heme oxygenase. *Free Radical Biology and Medicine*.

[64] Sulaiman M., Matta M. J., Sunderesan N. R., Gupta M. P., Periasamy M., Gupta M. (2010). Resveratrol, an activator of SIRT1, upregulates sarcoplasmic calcium ATPase and improves cardiac function in diabetic cardiomyopathy. *The American Journal of Physiology—Heart and Circulatory Physiology*.

[65] Zhang H., Morgan B., Potter B. J. (2010). Resveratrol improves left ventricular diastolic relaxation in type 2 diabetes by inhibiting oxidative/nitrative stress: in vivo demonstration with magnetic resonance imaging. *The American Journal of Physiology—Heart and Circulatory Physiology*.

[66] Crandall J. P., Oram V., Trandafirescu G. (2012). Pilot study of resveratrol in older adults with impaired glucose tolerance. *Journals of Gerontology—Series A: Biological Sciences and Medical Sciences*.

[67] Méndez-del Villar M., González-Ortiz M., Martínez-Abundis E., Pérez-Rubio K. G., Lizárraga-Valdez R. (2014). Effect of resveratrol administration on metabolic syndrome, insulin sensitivity, and insulin secretion. *Metabolic Syndrome and Related Disorders*.

[68] Chachay V. S., Macdonald G. A., Martin J. H. (2014). Resveratrol does not benefit patients with nonalcoholic fatty liver disease. *Clinical Gastroenterology and Hepatology*.

[69] Dash S., Xiao C., Morgantini C., Szeto L., Lewis G. F. (2013). High-dose resveratrol treatment for 2 weeks inhibits intestinal and hepatic lipoprotein production in overweight/obese men. *Arteriosclerosis, Thrombosis, and Vascular Biology*.

[70] Tomé-Carneiro J., Gonzálvez M., Larrosa M. (2013). Grape resveratrol increases serum adiponectin and downregulates inflammatory genes in peripheral blood mononuclear cells: a triple-blind, placebo-controlled, one-year clinical trial in patients with stable coronary artery disease. *Cardiovascular Drugs and Therapy*.

[71] De Groote D., van Belleghem K., Devire J., Van Brussel W., Mukaneza A., Amininejad L. (2012). Effect of the intake of resveratrol, resveratrol phosphate, and catechin-rich grape seed extract on markers of oxidative stress and gene expression in adult obese subjects. *Annals of Nutrition and Metabolism*.

[72] Hung L.-M., Su M.-J., Chen J.-K. (2004). Resveratrol protects myocardial ischemia-reperfusion injury through both NO-dependent and NO-independent mechanisms. *Free Radical Biology and Medicine*.

[73] Wang X.-B., Huang J., Zou J.-G. (2007). Effects of resveratrol on number and activity of endothelial progenitor cells from human peripheral blood. *Clinical and Experimental Pharmacology and Physiology*.

[74] Chen C.-J., Yu W., Fu Y.-C., Wang X., Li J.-L., Wang W. (2009). Resveratrol protects cardiomyocytes from hypoxia-induced apoptosis through the SIRT1-FoxO1 pathway. *Biochemical and Biophysical Research Communications*.

[75] Gurusamy N., Lekli I., Mukherjee S. (2010). Cardioprotection by resveratrol: a novel mechanism via autophagy involving the mTORC2 pathway. *Cardiovascular Research*.

[76] Kaga S., Zhan L., Matsumoto M., Maulik N. (2005). Resveratrol enhances neovascularization in the infarcted rat myocardium through the induction of thioredoxin-1, heme oxygenase-1 and vascular endothelial growth factor. *Journal of Molecular and Cellular Cardiology*.

[77] Gurusamy N., Ray D., Lekli I., Das D. K. (2010). Red wine antioxidant resveratrol-modified cardiac stem cells regenerate infarcted myocardium. *Journal of Cellular and Molecular Medicine*.

[78] Mukhopadhyay P., Mukherjee S., Ahsan K., Bagchi A., Pacher P., Das D. K. (2010). Restoration of altered MicroRNA expression in the ischemic heart with resveratrol. *PLoS ONE*.

[79] Shalwala M., Zhu S.-G., Das A., Salloum F. N., Xi L., Kukreja R. C. (2014). Sirtuin 1 (SIRT1) activation mediates sildenafil induced delayed cardioprotection against ischemia-reperfusion injury in mice. *PLoS ONE*.

[80] Kanamori H., Takemura G., Goto K. (2013). Resveratrol reverses remodeling in hearts with large, old myocardial infarctions through enhanced autophagy-activating AMP kinase pathway. *American Journal of Pathology*.

[81] Barseghian A., Gawande D., Bajaj M. (2011). Adiponectin and vulnerable atherosclerotic plaques. *Journal of the American College of Cardiology*.

[82] Maruyoshi H., Kojima S., Funahashi T. (2004). Adiponectin is inversely related to plasminogen activator inhibitor type 1 in patients with stable exertional angina. *Thrombosis and Haemostasis*.

[83] Zordoky B. N., Robertson I. M., Dyck J. R. (2014). Preclinical and clinical evidence for the role of resveratrol in the treatment of cardiovascular diseases. *Biochimica et Biophysica Acta*.

[84] Thandapilly S. J., Wojciechowski P., Behbahani J. (2010). Resveratrol prevents the development of pathological cardiac hypertrophy and contractile dysfunction in the SHR without lowering blood pressure. *American Journal of Hypertension*.

[85] Rimbaud S., Ruiz M., Piquereau J. (2011). Resveratrol improves survival, hemodynamics and energetics in a rat model of hypertension leading to heart failure. *PLoS ONE*.

[86] Wojciechowski P., Juric D., Louis X. L. (2010). Resveratrol arrests and regresses the development of pressure overload- but not volume overload-induced cardiac hypertrophy in rats. *Journal of Nutrition*.

[87] Ferroni P., Della-Morte D., Palmirotta R. (2011). Platinum-based compounds and risk for cardiovascular toxicity in the elderly: role of the antioxidants in chemoprevention. *Rejuvenation Research*.

[88] Gu X. S., Wang Z. B., Ye Z. (2014). Resveratrol, an activator of SIRT1, upregulates AMPK and improves cardiac function in heart failure. *Genetics and Molecular Research*.

[89] Yoshida Y., Shioi T., Izumi T. (2007). Resveratrol ameliorates experimental autoimmune myocarditis. *Circulation Journal*.

[90] Xuan W., Wu B., Chen C. (2012). Resveratrol improves myocardial ischemia and ischemic heart failure in mice by antagonizing the detrimental effects of fractalkine. *Critical Care Medicine*.

[91] Sung M. M., Das S. K., Levasseur J. (2015). Resveratrol treatment of mice with pressure-overload-induced heart failure improves diastolic function and cardiac energy metabolism. *Circulation: Heart Failure*.

[92] Clark D., Tuor U. I., Thompson R. (2012). Protection against recurrent stroke with resveratrol: endothelial protection. *PLoS ONE*.

[93] Simão F., Pagnussat A. S., Seo J. H. (2012). Pro-angiogenic effects of resveratrol in brain endothelial cells: nitric oxide-mediated regulation of vascular endothelial growth factor and metalloproteinases. *Journal of Cerebral Blood Flow and Metabolism*.

[94] Arrick D. M., Sun H., Patel K. P., Mayhan W. G. (2011). Chronic resveratrol treatment restores vascular responsiveness of cerebral arterioles in type 1 diabetic rats. *The American Journal of Physiology—Heart and Circulatory Physiology*.

[95] Huang S. S., Tsai M. C., Chih C. L., Hung L. M., Tsai S. K. (2001). Resveratrol reduction of infarct size in Long-Evans rats subjected to focal cerebral ischemia. *Life Sciences*.

[96] Chang H.-C., Tai Y.-T., Cherng Y.-G. (2014). Resveratrol attenuates high-fat diet-induced disruption of the blood-brain barrier and protects brain neurons from apoptotic insults. *Journal of Agricultural and Food Chemistry*.

[97] Ren J., Fan C., Chen N., Huang J., Yang Q. (2011). Resveratrol pretreatment attenuates cerebral ischemic injury by upregulating expression of transcription factor Nrf2 and HO-1 in Rats. *Neurochemical Research*.

[98] Della-Morte D., Dave K. R., DeFazio R. A., Bao Y. C., Raval A. P., Perez-Pinzon M. A. (2009). Resveratrol pretreatment protects rat brain from cerebral ischemic damage via a sirtuin 1-uncoupling protein 2 pathway. *Neuroscience*.

[99] Wightman E. L., Reay J. L., Haskell C. F., Williamson G., Dew T. P., Kennedy D. O. (2014). Effects of resveratrol alone or in combination with piperine on cerebral blood flow parameters and cognitive performance in human subjects: a randomised, double-blind, placebo-controlled, cross-over investigation. *British Journal of Nutrition*.

[100] Arafa M. H., Mohammad N. S., Atteia H. H., Abd-Elaziz H. R. (2014). Protective effect of resveratrol against doxorubicin-induced cardiac toxicity and fibrosis in male experimental rats. *Journal of Physiology and Biochemistry*.

[101] Wang G.-Y., Wang Y.-M., Zhang L.-N. (2007). Effect of resveratrol on heart function of rats with adriamycin-induced heart failure. *Zhongguo Zhong Yao Za Zhi*.

[102] Wang S., Song P., Zou M.-H. (2012). Inhibition of AMP-activated protein kinase *α* (AMPK*α*) by doxorubicin accentuates genotoxic stress and cell death in mouse embryonic fibroblasts and cardiomyocytes: role of p53 and SIRT1. *The Journal of Biological Chemistry*.

[103] Zhang Y., Liu Y., Wang T. (2006). Resveratrol, a natural ingredient of grape skin: antiarrhythmic efficacy and ionic mechanisms. *Biochemical and Biophysical Research Communications*.

[104] Chen Y. R., Yi F. F., Li X. Y. (2008). Resveratrol attenuates ventricular arrhythmias and improves the long-term survival in rats with myocardial infarction. *Cardiovascular Drugs and Therapy*.

[105] Baczko I., Liknes D., Yang W. (2014). Characterization of a novel multifunctional resveratrol derivative for the treatment of atrial fibrillation. *British Journal of Pharmacology*.

[106] Akhlaghi M., Bandy B. (2010). Dietary broccoli sprouts protect against myocardial oxidative damage and cell death during ischemia-reperfusion. *Plant Foods for Human Nutrition*.

[107] Kataya H. A. H., Hamza A. A. (2008). Red cabbage (*Brassica oleracea*) ameliorates diabetic nephropathy in rats. *Evidence-Based Complementary and Alternative Medicine*.

[108] Kim S. Y., Yoon S., Kwon S. M., Park K. S., Lee-Kim Y. C. (2008). Kale Juice improves coronary artery disease risk factors in hypercholesterolemic men. *Biomedical and Environmental Sciences*.

[109] Domínguez-Perles R., Mena P., García-Viguera C., Moreno D. A. (2014). Brassica foods as a dietary source of vitamin C: a review. *Critical Reviews in Food Science and Nutrition*.

[110] Mithen R., Bennett R., Marquez J. (2010). Glucosinolate biochemical diversity and innovation in the Brassicales. *Phytochemistry*.

[111] Houghton C. A., Fassett R. G., Coombes J. S. (2013). Sulforaphane: translational research from laboratory bench to clinic. *Nutrition Reviews*.

[112] Conaway C. C., Getahun S. M., Liebes L. L. (2000). Disposition of glucosinolates and sulforaphane in humans after ingestion of steamed and fresh broccoli. *Nutrition and Cancer*.

[113] Vermeulen M., van Rooijen H. J. M., Vaes W. H. J. (2003). Analysis of isothiocyanate mercapturic acids in urine: a biomarker for cruciferous vegetable intake. *Journal of Agricultural and Food Chemistry*.

[114] Ritz S. A., Wan J., Diaz-Sanchez D. (2007). Sulforaphane-stimulated phase II enzyme induction inhibits cytokine production by airway epithelial cells stimulated with diesel extract. *The American Journal of Physiology—Lung Cellular and Molecular Physiology*.

[115] Oak M.-H., Bedoui J. E., Madeira S. V. F., Chalupsky K., Schini-Kerth V. B. (2006). Delphinidin and cyanidin inhibit PDGF_AB_-induced VEGF release in vascular smooth muscle cells by preventing activation of p38 MAPK and JNK. *British Journal of Pharmacology*.

[116] Palfi A., Bartha E., Copf L. (2009). Alcohol-free red wine inhibits isoproterenol-induced cardiac remodeling in rats by the regulation of Akt1 and protein kinase C *α*/*β* II. *Journal of Nutritional Biochemistry*.

[117] Rubattu S., di Castro S., Cotugno M. (2015). Protective effects of Brassica oleracea sprouts extract toward renal damage in high-salt-fed SHRSP: role of AMPK/PPAR*α*/UCP2 axis. *Journal of Hypertension*.

[118] Zakkar M., van der Heiden K., Luong L. A. (2009). Activation of Nrf2 in endothelial cells protects arteries from exhibiting a proinflammatory state. *Arteriosclerosis, Thrombosis, and Vascular Biology*.

[119] Murashima M., Watanabe S., Zhuo X.-G., Uehara M., Kurashige A. (2004). Phase 1 study of multiple biomarkers for metabolism and oxidative stress after one-week intake of broccoli sprouts. *BioFactors*.

[120] Wu L., Ashraf M. H. N., Facci M. (2004). Dietary approach to attenuate oxidative stress, hypertension, and inflammation in the cardiovascular system. *Proceedings of the National Academy of Sciences of the United States of America*.

[121] Noyan-Ashraf M. H., Wu L., Wang R., Juurlink B. H. J. (2006). Dietary approaches to positively influence fetal determinants of adult health. *The FASEB Journal*.

[122] Rechner A. R., Kroner C. (2005). Anthocyanins and colonic metabolites of dietary polyphenols inhibit platelet function. *Thrombosis Research*.

[123] Garcia-Alonso M., Minihane A.-M., Rimbach G., Rivas-Gonzalo J. C., de Pascual-Teresa S. (2009). Red wine anthocyanins are rapidly absorbed in humans and affect monocyte chemoattractant protein 1 levels and antioxidant capacity of plasma. *Journal of Nutritional Biochemistry*.

[124] Bell D. R., Gochenaur K. (2006). Direct vasoactive and vasoprotective properties of anthocyanin-rich extracts. *Journal of Applied Physiology*.

[125] Toufektsian M.-C., de Lorgeril M., Nagy N. (2008). Chronic dietary intake of plant-derived anthocyanins protects the rat heart against ischemia-reperfusion injury. *Journal of Nutrition*.

[126] Xia M., Ling W., Zhu H. (2007). Anthocyanin prevents CD40-activated proinflammatory signaling in endothelial cells by regulating cholesterol distribution. *Arteriosclerosis, Thrombosis, and Vascular Biology*.

[127] Hämäläinen M., Nieminen R., Vuorela P., Heinonen M., Moilanen E. (2007). Anti-inflammatory effects of flavonoids: genistein, kaempferol, quercetin, and daidzein inhibit STAT-1 and NF-*κ*B activations, whereas flavone, isorhamnetin, naringenin, and pelargonidin inhibit only NF-*κ*B activation along with their inhibitory effect on iNOS expression and NO production in activated macrophages. *Mediators of Inflammation*.

[128] Scarabelli T. M., Mariotto S., Abdel-Azeim S. (2009). Targeting STAT1 by myricetin and delphinidin provides efficient protection of the heart from ischemia/reperfusion-induced injury. *FEBS Letters*.

[129] Jia Y., Kim J.-Y., Jun H.-J. (2013). Cyanidin is an agonistic ligand for peroxisome proliferator-activated receptor-alpha reducing hepatic lipid. *Biochimica et Biophysica Acta—Molecular and Cell Biology of Lipids*.

[130] Clarke J. D., Riedl K., Bella D., Schwartz S. J., Stevens J. F., Ho E. (2011). Comparison of isothiocyanate metabolite levels and histone deacetylase activity in human subjects consuming broccoli sprouts or broccoli supplement. *Journal of Agricultural and Food Chemistry*.

[131] Cramer J. M., Jeffery E. H. (2011). Sulforaphane absorption and excretion following ingestion of a semi-purified broccoli powder rich in glucoraphanin and broccoli sprouts in healthy men. *Nutrition and Cancer*.

[132] Bahadoran Z., Mirmiran P., Hosseinpanah F., Rajab A., Asghari G., Azizi F. (2012). Broccoli sprouts powder could improve serum triglyceride and oxidized LDL/LDL-cholesterol ratio in type 2 diabetic patients: a randomized double-blind placebo-controlled clinical trial. *Diabetes Research and Clinical Practice*.

[133] Jennings A., Welch A. A., Fairweather-Tait S. J. (2012). Higher anthocyanin intake is associated with lower arterial stiffness and central blood pressure in women. *The American Journal of Clinical Nutrition*.

[134] Curtis P. J., Kroon P. A., Hollands W. J. (2009). Cardiovascular disease risk biomarkers and liver and kidney function are not altered in postmenopausal women after ingesting an elderberry extract rich in anthocyanins for 12 weeks. *Journal of Nutrition*.

[135] Aggarwal B. B., Harikumar K. B. (2009). Potential therapeutic effects of curcumin, the anti-inflammatory agent, against neurodegenerative, cardiovascular, pulmonary, metabolic, autoimmune and neoplastic diseases. *International Journal of Biochemistry and Cell Biology*.

[136] Wongcharoen W., Phrommintikul A. (2009). The protective role of curcumin in cardiovascular diseases. *International Journal of Cardiology*.

[137] Duan W., Yang Y., Yan J. (2012). The effects of curcumin post-treatment against myocardial ischemia and reperfusion by activation of the JAK2/STAT3 signaling pathway. *Basic Research in Cardiology*.

[138] Pu Y., Zhang H., Wang P. (2013). Dietary curcumin ameliorates aging-related cerebrovascular dysfunction through the ampk/uncoupling protein 2 pathway. *Cellular Physiology and Biochemistry*.

[139] Wu J., Li Q., Wang X. (2013). Neuroprotection by curcumin in ischemic brain injury involves the Akt/Nrf2 pathway. *PLoS ONE*.

[140] Deng Z.-H., Liao J., Zhang J.-Y. (2013). Localized leptin release may be an important mechanism of curcumin action after acute ischemic injuries. *Journal of Trauma and Acute Care Surgery*.

[141] Kim Y. S., Ahn Y., Hong M. H. (2007). Curcumin attenuates inflammatory responses of TNF-alpha-stimulated human endothelial cells. *Journal of Cardiovascular Pharmacology*.

[142] Sahebkar A. (2014). Are curcuminoids effective C-reactive protein-lowering agents in clinical practice? Evidence from a meta-analysis. *Phytotherapy Research*.

[143] Parodi F. E., Mao D., Ennis T. L., Pagano M. B., Thompson R. W. (2006). Oral administration of diferuloylmethane (curcumin) suppresses proinflammatory cytokines and destructive connective tissue remodeling in experimental abdominal aortic aneurysms. *Annals of Vascular Surgery*.

[144] Pan Y., Zhu G., Wang Y. (2013). Attenuation of high-glucose-induced inflammatory response by a novel curcumin derivative B06 contributes to its protection from diabetic pathogenic changes in rat kidney and heart. *Journal of Nutritional Biochemistry*.

[145] Manikandan P., Sumitra M., Aishwarya S., Manohar B. M., Lokanadam B., Puvanakrishnan R. (2004). Curcumin modulates free radical quenching in myocardial ischaemia in rats. *The International Journal of Biochemistry & Cell Biology*.

[146] Farhangkhoee H., Khan Z. A., Chen S., Chakrabarti S. (2006). Differential effects of curcumin on vasoactive factors in the diabetic rat heart. *Nutrition and Metabolism*.

[147] Motterlini R., Foresti R., Bassi R., Green C. J. (2000). Curcumin, an antioxidant and anti-inflammatory agent, induces heme oxygenase-1 and protects endothelial cells against oxidative stress. *Free Radical Biology and Medicine*.

[148] Trujillo J., Granados-Castro L. F., Zazueta C., Andérica-Romero A. C., Chirino Y. I., Pedraza-Chaverrí J. (2014). Mitochondria as a target in the therapeutic properties of curcumin. *Archiv der Pharmazie*.

[149] Yang X., Thomas D. P., Zhang X. (2006). Curcumin inhibits platelet-derived growth factor-stimulated vascular smooth muscle cell function and injury-induced neointima formation. *Arteriosclerosis, Thrombosis, and Vascular Biology*.

[150] Chen H.-W., Huang H.-C. (1998). Effect of curcumin on cell cycle progression and apoptosis in vascular smooth muscle cells. *British Journal of Pharmacology*.

[151] Hua Y., Dolence J., Ramanan S., Ren J., Nair S. (2013). Bisdemethoxycurcumin inhibits PDGF-induced vascular smooth muscle cell motility and proliferation. *Molecular Nutrition and Food Research*.

[152] Sumbilla C., Lewis D., Hammerschmidt T., Inesi G. (2002). The slippage of the Ca^2+^ pump and its control by anions and curcumin in skeletal and cardiac sarcoplasmic reticulum. *The Journal of Biological Chemistry*.

[153] Zingg J.-M., Hasan S. T., Meydani M. (2013). Molecular mechanisms of hypolipidemic effects of curcumin. *BioFactors*.

[154] Quiles J. L., Mesa M. D., Ramírez-Tortosa C. L. (2002). Curcuma longa extract supplementation reduces oxidative stress and attenuates aortic fatty streak development in rabbits. *Arteriosclerosis, Thrombosis, and Vascular Biology*.

[155] Nirmala C., Puvanakrishnan R. (1996). Protective role of curcumin against isoproterenol induced myocardial infarction in rats. *Molecular and Cellular Biochemistry*.

[156] Wang N.-P., Wang Z.-F., Tootle S., Philip T., Zhao Z.-Q. (2012). Curcumin promotes cardiac repair and ameliorates cardiac dysfunction following myocardial infarction. *British Journal of Pharmacology*.

[157] Yeh C.-H., Chen T.-P., Wu Y.-C., Lin Y.-M., Jing Lin P. (2005). Inhibition of NF*κ*B activation with curcumin attenuates plasma inflammatory cytokines surge and cardiomyocytic apoptosis following cardiac ischemia/reperfusion. *Journal of Surgical Research*.

[158] Morimoto T., Sunagawa Y., Kawamura T. (2008). The dietary compound curcumin inhibits p300 histone acetyltransferase activity and prevents heart failure in rats. *Journal of Clinical Investigation*.

[159] Venkatesan N. (1998). Curcumin attenuation of acute adriamycin myocardial toxicity in rats. *British Journal of Pharmacology*.

[160] Chen T.-H., Yang Y.-C., Wang J.-C., Wang J.-J. (2013). Curcumin treatment protects against renal ischemia and reperfusion injury-induced cardiac dysfunction and myocardial injury. *Transplantation Proceedings*.

[161] Kakkar V., Muppu S. K., Chopra K., Kaur I. P. (2013). Curcumin loaded solid lipid nanoparticles: an efficient formulation approach for cerebral ischemic reperfusion injury in rats. *European Journal of Pharmaceutics and Biopharmaceutics*.

[162] Ahmad N., Umar S., Ashafaq M. (2013). A comparative study of PNIPAM nanoparticles of curcumin, demethoxycurcumin, and bisdemethoxycurcumin and their effects on oxidative stress markers in experimental stroke. *Protoplasma*.

[163] Funk J. L., Frye J. B., Davis-Gorman G. (2013). Curcuminoids limit neutrophil-mediated reperfusion injury in experimental stroke by targeting the endothelium. *Microcirculation*.

[164] Soni K. B., Kuttan R. (1992). Effect of oral curcumin administration on serum peroxides and cholesterol levels in human volunteers. *Indian Journal of Physiology and Pharmacology*.

[165] Alwi I., Santoso T., Suyono S. (2008). The effect of curcumin on lipid level in patients with acute coronary syndrome. *Acta Medica Indonesiana*.

[166] Chuengsamarn S., Rattanamongkolgul S., Phonrat B., Tungtrongchitr R., Jirawatnotai S. (2014). Reduction of atherogenic risk in patients with type 2 diabetes by curcuminoid extract: a randomized controlled trial. *Journal of Nutritional Biochemistry*.

[167] Sahebkar A. (2014). A systematic review and meta-analysis of randomized controlled trials investigating the effects of curcumin on blood lipid levels. *Clinical Nutrition*.

[168] Akazawa N., Choi Y., Miyaki A. (2012). Curcumin ingestion and exercise training improve vascular endothelial function in postmenopausal women. *Nutrition Research*.

[169] Pan C. J., Tang J. J., Shao Z. Y., Wang J., Huang N. (2007). Improved blood compatibility of rapamycin-eluting stent by incorporating curcumin. *Colloids and Surfaces B: Biointerfaces*.

[170] Liu Y.-X., Xiao C.-L., Wang Y.-X. (2012). Synthesis, structure-activity relationship and in vitro anti-mycobacterial evaluation of 13-n-octylberberine derivatives. *European Journal of Medicinal Chemistry*.

[171] Nishino H., Kitagawa K., Fujiki H., Iwashima A. (1986). Berberine sulfate inhibits tumor-promoting activity of teleocidin in two-stage carcinogenesis on mouse skin. *Oncology*.

[172] Yao J., Kong W., Jiang J. (2013). Learning from berberine: treating chronic diseases through multiple targets. *Science China Life Sciences*.

[173] Lau C. W., Yao X. Q., Chen Z. Y., Ko W. H., Huang Y. (2001). Cardiovascular actions of berberine. *Cardiovascular Drug Reviews*.

[174] Wang Q., Zhang M., Liang B., Shirwany N., Zhu Y., Zou M.-H. (2011). Activation of AMP-activated protein kinase is required for berberine-induced reduction of atherosclerosis in mice: the role of uncoupling protein 2. *PLoS ONE*.

[175] Sarna L. K., Wu N., Hwang S.-Y., Siow Y. L., Karmin O. (2010). Berberine inhibits NADPH oxidase mediated superoxide anion production in macrophages. *Canadian Journal of Physiology and Pharmacology*.

[176] Mo C., Wang L., Zhang J. (2014). The crosstalk between Nrf2 and AMPK signal pathways is important for the anti-inflammatory effect of berberine in LPS-stimulated macrophages and endotoxin-shocked mice. *Antioxidants and Redox Signaling*.

[177] Jeong H. W., Hsu K. C., Lee J.-W. (2009). Berberine suppresses proinflammatory responses through AMPK activation in macrophages. *American Journal of Physiology—Endocrinology and Metabolism*.

[178] Xie X., Chang X., Chen L. (2013). Berberine ameliorates experimental diabetes-induced renal inflammation and fibronectin by inhibiting the activation of RhoA/ROCK signaling. *Molecular and Cellular Endocrinology*.

[179] Feng A.-W., Gao W., Zhou G.-R. (2012). Berberine ameliorates COX-2 expression in rat small intestinal mucosa partially through PPARgamma pathway during acute endotoxemia. *International Immunopharmacology*.

[180] Xiao M., Men L. N., Xu M. G., Wang G. B., Lv H. T., Liu C. (2014). Berberine protects endothelial progenitor cell from damage of TNF-*α* via the PI3K/AKT/eNOS signaling pathway. *European Journal of Pharmacology*.

[181] Wang Y., Huang Y., Lam K. S. L. (2009). Berberine prevents hyperglycemia-induced endothelial injury and enhances vasodilatation via adenosine monophosphate-activated protein kinase and endothelial nitric oxide synthase. *Cardiovascular Research*.

[182] Lee S., Lim H.-J., Park H.-Y., Lee K.-S., Park J.-H., Jang Y. (2006). Berberine inhibits rat vascular smooth muscle cell proliferation and migration in vitro and improves neointima formation after balloon injury in vivo. Berberine improves neointima formation in a rat model. *Atherosclerosis*.

[183] Zimetti F., Adorni M. P., Ronda N., Gatti R., Bernini F., Favari E. (2015). The natural compound berberine positively affects macrophage functions involved in atherogenesis. *Nutrition, Metabolism and Cardiovascular Diseases*.

[184] Derosa G., Maffioli P., Cicero A. F. G. (2012). Berberine on metabolic and cardiovascular risk factors: an analysis from preclinical evidences to clinical trials. *Expert Opinion on Biological Therapy*.

[185] Kong W. J., Wei J., Abidi P. (2004). Berberine is a novel cholesterol-lowering drug working through a unique mechanism distinct from statins. *Nature Medicine*.

[186] Li H., Dong B., Park S. W., Lee H.-S., Chen W., Liu J. (2009). Hepatocyte nuclear factor 1*α* plays a critical role in PCSK9 gene transcription and regulation by the natural hypocholesterolemic compound berberine. *The Journal of Biological Chemistry*.

[187] Jia Y.-J., Xu R.-X., Sun J., Tang Y., Li J.-J. (2014). Enhanced circulating PCSK9 concentration by berberine through SREBP-2 pathway in high fat diet-fed rats. *Journal of Translational Medicine*.

[188] Lau C.-W., Yao X.-Q., Chen Z.-Y., Ko W.-H., Huang Y. (2001). Cardiovascular actions of berberine. *Cardiovascular Drug Reviews*.

[189] Wang L.-H., Li X.-L., Li Q. (2012). Berberine alleviates ischemic arrhythmias via recovering depressed I(to) and I(Ca) currents in diabetic rats. *Phytomedicine*.

[190] Rodriguez-Menchaca A., Ferrer-Villada T., Lara J., Fernandez D., Navarro-Polanco R. A., Sanchez-Chapula J. A. (2006). Block of hERG channels by berberine: mechanisms of voltage- and state-dependence probed with site-directed mutant channels. *Journal of Cardiovascular Pharmacology*.

[191] Neto F. R. (1993). Electropharmacological effects of berberine on canine cardiac Purkinje fibres and ventricular muscle and atrial muscle of the rabbit. *British Journal of Pharmacology*.

[192] Liu J.-C., Chan P., Chen Y.-J., Tomlinson B., Hong S.-F., Cheng J.-T. (1999). The antihypertensive effect of the berberine derivative 6-protoberberine in spontaneously hypertensive rats. *Pharmacology*.

[193] Li M.-H., Zhang Y.-J., Yu Y.-H. (2014). Berberine improves pressure overload-induced cardiac hypertrophy and dysfunction through enhanced autophagy. *European Journal of Pharmacology*.

[194] Zhang T., Yang S., Du J. (2015). Protective effects of berberine on isoproterenol-induced acute myocardial ischemia in rats through regulating hmgb1-tlr4 axis. *Evidence-Based Complementary and Alternative Medicine*.

[195] Liu L., Liu J., Huang Z. (2015). Berberine improves endothelial function by inhibiting endoplasmic reticulum stress in the carotid arteries of spontaneously hypertensive rats. *Biochemical and Biophysical Research Communications*.

[196] Zhang Y.-J., Yang S.-H., Li M.-H. (2014). Berberine attenuates adverse left ventricular remodeling and cardiac dysfunction after acute myocardial infarction in rats: role of autophagy. *Clinical and Experimental Pharmacology and Physiology*.

[197] Chen K., Li G., Geng F. (2014). Berberine reduces ischemia/reperfusion-induced myocardial apoptosis via activating AMPK and PI3K-Akt signaling in diabetic rats. *Apoptosis*.

[198] Zhou X.-Q., Zeng X.-N., Kong H., Sun X.-L. (2008). Neuroprotective effects of berberine on stroke models in vitro and in vivo. *Neuroscience Letters*.

[199] Dong H., Wang N., Zhao L., Lu F. (2012). Berberine in the treatment of type 2 diabetes mellitus: a systemic review and meta-analysis. *Evidence-Based Complementary and Alternative Medicine*.

[200] Zeng X.-H., Zeng X.-J., Li Y.-Y. (2003). Efficacy and safety of berberine for congestive heart failure secondary to ischemic or idiopathic dilated cardiomyopathy. *American Journal of Cardiology*.

[201] Huang W. (1990). Ventricular tachyarrhythmias treated with berberine. *Zhonghua Xin Xue Guan Bing Za Zhi*.

[202] Jiang C., Kuang Y. (1998). Therapeutic efficacy of berberine in 32 arrhythmic patients. *Zhong Guo Zhong Xi Yi Jie He Ji Jiu Za Zhi*.

[203] Dong H., Zhao Y., Zhao L., Lu F. (2013). The effects of berberine on blood lipids: a systemic review and meta-analysis of randomized controlled trials. *Planta Medica*.

[204] Liu L., Yu Y.-L., Yang J.-S. (2010). Berberine suppresses intestinal disaccharidases with beneficial metabolic effects in diabetic states, evidences from in vivo and in vitro study. *Naunyn-Schmiedeberg's Archives of Pharmacology*.

[205] Affuso F., Ruvolo A., Micillo F., Saccà L., Fazio S. (2010). Effects of a nutraceutical combination (berberine, red yeast rice and policosanols) on lipid levels and endothelial function randomized, double-blind, placebo-controlled study. *Nutrition, Metabolism and Cardiovascular Diseases*.

[206] Lan J., Zhao Y., Dong F. (2015). Meta-analysis of the effect and safety of berberine in the treatment of type 2 diabetes mellitus, hyperlipemia and hypertension. *Journal of Ethnopharmacology*.

